# Nurse-perpetrated abuse in Japanese psychiatric hospitals: a cross-sectional study of prevalence and correlates

**DOI:** 10.3389/fpsyt.2025.1624859

**Published:** 2025-11-24

**Authors:** Kei Matoba, So Yayama, Taiki Teshima, Akiko Miki

**Affiliations:** Faculty of Nursing, Psychiatric and Mental Health Nursing, Kansai Medical University, Hirakata, Japan

**Keywords:** physical abuse, psychiatric hospitals, nurses, violence, prevalence, coercion

## Abstract

**Introduction:**

The abuse of psychiatric inpatients in psychiatric hospitals is a global concern. Although the prevalence may be underestimated due to underreporting rates of physical abuse and restraint have been reported at 6% and 40%, respectively. However, data on this issue are scarce, where the picture is further complicated by the difficulty in defining “abuse” within a context where certain coercive measures are legally permitted. This study aimed to investigate the prevalence of such abuse and its psychological and workplace-related correlates.

**Methods:**

We conducted a cross-sectional survey of 203 nursing staff working in eight psychiatric hospitals located in both rural and urban areas of Japan. A self-administered, web-based questionnaire battery assessed demographic characteristics, workplace violence, inappropriate and abusive behaviors, moral sensitivity, ethical climate, attitudes toward recovery, job stressors, and moral distress.

**Results:**

The prevalence of respondents who reported experiencing at least one of the 32 abusive behaviors of interest was 87.1%, with ignoring or rejecting patients being the most common form. Logistic regression analysis revealed that experience of workplace violence and more recovery-oriented attitudes were significantly associated with higher odds of engaging in abusive behaviors (adjusted odds ratios: 3.37 and 1.17, respectively), whereas greater moral sensitivity and longer clinical experience were inversely associated (adjusted odds ratios: 0.92 and 0.95, respectively).

**Discussion:**

These findings indicate a relatively high prevalence of nurse-to-patient abuse in Japanese psychiatric hospitals and highlight the complex interplay of individual and workplace factors in such behaviors. This evidence may serve as a foundation for the development of targeted interventions aimed at preventing abuse in psychiatric care settings.

## 1 Introduction

Psychiatric inpatients are among the most vulnerable patient groups, as severe mental illness and institutional power imbalances can leave them open to mistreatment. Unfortunately, abuse in psychiatric hospitals continues to be a major global concern. The World Health Organization (WHO) and the Office of the United Nations High Commissioner for Human Rights (OHCHR) jointly published Mental Health, Human Rights and Legislation: Guidance and Practice (2023), which documented persistent violations such as arbitrary detention, prolonged seclusion, physical restraint, and forced treatment in psychiatric facilities across various countries (1). These coercive practices are often justified as therapeutic interventions but may infringe upon patients’ dignity and autonomy when applied inappropriately. Previous research similarly highlights instances of patient abuse and neglect in inpatient psychiatry, even in recent years (2), however, empirical data on the scope of the problem remain limited. Many incidents of abuse likely go unreported; for example, professional sexual misconduct in healthcare is believed to be widely underreported because of problematic reporting processes (3). As a result, the prevalence of abuse is hard to ascertain. Nevertheless, patient surveys confirm that abuse does occur: in one 10-year follow-up study of patients with psychosis, 6% reported experiencing physical abuse by hospital staff during hospitalization (4). High rates of coercion were also observed in that cohort (i.e. 62% of patients were involuntarily admitted, and 40% were restrained) (4), underscoring how common forceful interventions are. These findings suggest that although overt abuse by staff may be under-recognized, coercive or distressing practices are frequent and can easily cross ethical lines.

A key challenge in mental health care is defining and preventing “abuse” within a context where certain coercive measures are legally and clinically sanctioned. In general, abuse of patients can manifest in various forms, including not only physical violence but also psychological mistreatment, sexual exploitation, financial exploitation, and neglect of care. Such abuse may result from individual misconduct or be embedded in routine clinical practices. In Japan, coercive measures such as involuntary hospitalization, seclusion, and physical restraint are legally permitted under the Mental Health and Welfare Act when patients are deemed at risk of harming themselves or others. These interventions are intended to ensure safety but, when applied excessively, for prolonged periods, or without sufficient justification or respect for patient dignity, they may constitute abusive practices. This concern is not unique to Japan but has been raised internationally as well. The WHO notes that coercive or substandard institutional practices—such as forced admission, inappropriate use of restraints, and unsanitary living conditions—remain widespread and constitute violations of patients’ rights (1). Therefore, beyond direct physical, psychological or sexual abuse by staff, there are organizational and institutional dimensions of abuse. A rigid, custodial hospital milieu that overly restricts autonomy or dignity can itself be abusive. Psychiatric nursing illustrates the ethical complexities involved in balancing autonomy and safety. The line between therapeutic coercion and abuse can be thin. For example, the use of physical restraint or seclusion is intended to prevent harm, but if applied excessively or without respect for the patient becomes an act of violence or humiliation (6, 7). Ensuring care is trauma-informed and respectful is crucial, as even well-intended interventions can traumatize patients or erode their trust (8). In this context, the ethical climate of the care setting plays a major role. A positive ethical climate and a recovery-oriented approach—emphasizing patient empowerment and hope—might protect against abusive practices (9), whereas a stressful or punitive work environment could increase the risk of staff mistreating patients (10). A nurse’s moral sensitivity and personal values are also relevant: nurses with higher ethical awareness and empathy may be less likely to engage in or tolerate abuse, whereas those who are desensitized or burned out might rationalize harsh treatment (11). Therefore, we consider the prevention of abuse in psychiatric settings to be not merely a matter of individual ethics, but also a structural issue rooted in organizational policies and institutional culture.

Despite growing conceptual attention to the ethical and institutional dimensions of abuse, empirical research remains limited, especially from the perspective of healthcare staff such as nurses (5). While abuse in mental health settings is widely acknowledged, most existing knowledge is based on patients’ reports or case investigations, rather than data from staff involved in or witnesses to such behaviors. In other caregiving domains, such as nursing homes, staff self-report surveys have been used to quantify abuse and identify risk factors in caregivers (12), but comparable data are virtually non-existent for psychiatric hospitals. This lack of “perpetrator-side” data represents a critical gap in our understanding of the full dynamics of abuse. Furthermore, the mechanisms and risk factors of abusive conduct in psychiatric nursing remain largely speculative. Previous research has suggested that caregiver stress or impaired mental health can contribute to patient abuse (13), but little empirical evidence exists in psychiatric settings. We do not yet know whether, for instance, a nurse’s own experience of being assaulted or threatened by patients (a common occurrence in the psychiatry setting) is associated with a higher likelihood of that nurse later engaging in aggressive or abusive behaviors. Similarly, concepts like moral distress—the psychological stress nurses feel when they cannot act according to their ethical beliefs—might play a role in the erosion of compassionate care, potentially leading to callous or harmful behaviors (13, 14). Environmental factors are also plausible contributors: a poor ethical climate on the ward (where unethical behavior is ignored or normative) could facilitate abuse, whereas strong leadership and a culture of ethical accountability might curb it (15, 16). Furthermore, nurses who adopt a recovery-oriented model—prioritizing patient choice, hope, and rehabilitation—may manage conflicts more respectfully than those with a more authoritarian or pessimistic outlook, who may be more prone to resort to coercive or punitive measures (17). To date, however, no study has rigorously examined how such psychological and workplace factors correlate with instances of nurse-to-patient abuse. Addressing this gap is crucial for developing evidence-based strategies to prevent mistreatment in psychiatric care.

The present study aims to clarify the prevalence of abuse perpetrated by nurses in psychiatric hospitals, as well as to identify factors associated with abusive behaviors. The findings are expected to provide evidence to inform prevention strategies that aim to safeguard vulnerable patients, enhance therapeutic environments, and uphold the human rights and dignity of individuals receiving mental health care. These insights may assist managers, policymakers, and practitioners in implementing evidence-based measures to eliminate abuse and promote safer, more ethical inpatient psychiatric care.

## 2 Materials and methods

### 2.1 Study design and setting

We employed a cross-sectional study design to investigate the frequency of abuse by nursing staff within psychiatric hospitals and the associated factors. The study adhered to the Strengthening the Reporting of Observational Studies in Epidemiology (STROBE) guidelines. The research was conducted between November and December 2024 using a web-based, self-administered questionnaire. The online platform used for data collection ensured participant anonymity and confidentiality. Eligible participants were nursing staff working in psychiatric hospitals in Japan. The inclusion criteria were as follows: 1) currently employed as a registered nurse, assistant nurse, or nurse aide; 2) working in inpatient psychiatric wards; and 3) not serving in a managerial or supervisory position.

### 2.2 Data collection

This study involved nursing staff working in eight psychiatric hospitals recruited from regions across Japan, including both urban and rural regions (i.e. Tohoku, Kanto, Kinki, and Kyushu). These hospitals were selected either because of their participation in academic conferences on abuse prevention or their existing relationships with the first author. All participating hospitals were actively engaged in abuse prevention initiatives, which was a prerequisite given the sensitivity of the topic and the legal obligations surrounding abuse reporting.

Initial contact was made with each hospital’s director of nursing to request participation. After confirming which psychiatric wards could potentially cooperate, invitation letters were distributed in numbers corresponding to the estimated numbers of eligible staff. These letters included a QR code linked to the survey. Participation was entirely voluntary. A total of 685 survey invitations containing a QR code were distributed, and 209 responses (response rate, 30%) were received. After removing 6 duplicate entries, 203 unique responses were included in the analysis.

### 2.3 Measures

#### 2.3.1 Overview

The questionnaire consisted of the following components: demographic characteristics, workplace violence, abusive behaviors, the Japanese version of the Moral Sensitivity Questionnaire 2018 (J-MSQ2018), the Japanese version of the Hospital Ethical Climate Survey (J-HECS), the Japanese version of the Recovery Attitude Questionnaire (RAQ), the Psychiatric Nurse Job Stressor Scale (PNJSS), and the Moral Distress Scale for Psychiatric Nurses (MDS-P).

#### 2.3.2 Demographic characteristics

Demographic characteristics included sex, age, qualification (registered nurse, enrolled nurse, or nurse aide), employment status (full-time or part-time), years of clinical experience, years of psychiatric nursing experience, ward function (psychiatric emergency and acute care ward, general psychiatric ward, psychiatric long-term care ward, or other), ward type (locked, open, or mixed), and experience of workplace violence. Experience of workplace violence was assessed using a single item asking whether participants had experienced any physical, psychological, sexual, or other form of violence from patients during the past 12 months (response options: Yes/No).

#### 2.3.3 Abuse measures

To assess abusive behaviors by nursing staff, we developed a 32-item original questionnaire. The items were derived from three primary sources: 1) the Psychiatric Experiences Questionnaire (18), which categorizes experiences such as coercion, verbal abuse, and institutional procedures; 2) findings from a Japanese cross-sectional survey investigating abusive behaviors and ethical practices among psychiatric nurses (19); and 3) qualitative data obtained from preliminary interviews conducted by the present research team. The interview data were collected from psychiatric nursing managers, who shared their perceptions and experiences regarding abusive behaviors and their contributing factors in clinical practice. On the basis of insights from these interviews, we generated and refined items to reflect real-world ethical challenges faced by nurses. Before data collection, all items were reviewed by all authors to ensure their clarity and comprehensibility.

The items encompass a wide range of inappropriate behaviors, including physical, psychological, sexual, and economic abuse, neglect, and human rights violations. Among these, “use of excessive physical force during restraint” referred to situations in which nurses applied unnecessary or disproportionate force when restraining a patient. In Japan, the Mental Health and Welfare Act permits physical restraint only when necessary to prevent imminent harm to the patient or others, and requires medical authorization and documentation. Participants were asked to indicate the frequency with which they had engaged in each behavior over the past 12 months using a six-point Likert scale, where 1 = Never, 2 = Once, 3 = Rarely (once every 6 months), 4 = Occasionally (every 2–3 months), 5 = Monthly, and 6 = Weekly or more. They were instructed to choose the number that best reflected their experience, even if it was not an exact match. In the present study, the 32-item abuse questionnaire demonstrated good internal consistency (Cronbach’s α = 0.90, N = 203).

#### 2.3.4 Moral sensitivity

Moral sensitivity was assessed using the J-MSQ2018 (20), developed on the basis of the revised Moral Sensitivity Questionnaire (21). The original Moral Sensitivity Questionnaire consisted of nine items assessing three domains: moral strength, moral responsibility, and moral burden. The first Japanese version demonstrated structural validity but showed low internal consistency in the moral responsibility subscale (Cronbach’s α = 0.144), prompting the development of the J-MSQ2018. The J-MSQ2018 contains nine items—three per domain—rated on a six-point Likert scale ranging from “not at all applicable” to “very applicable.” In the present study, the Cronbach’s α coefficients were 0.835 for the total score, 0.801 for moral strength, 0.627 for moral burden, and 0.766 for moral responsibility.

#### 2.3.5 Ethical climate

Ethical climate was measured using the Japanese version of the J-HECS, developed by Inagaki et al. (22) on the basis of the original HECS (23). The scale evaluates nurses’ perceptions of the ethical climate across five domains: peers, patients, managers, the hospital, and physicians. The J-HECS includes 18 items rated on a five-point Likert scale ranging from 1 (“not at all true”) to 5 (“almost always true”). In the present study, the Cronbach’s α coefficients were 0.938 for the total score, 0.843 for peers, 0.931 for managers, 0.872 for the hospital, 0.739 for patients, and 0.836 for physicians.

#### 2.3.6 Recovery attitudes

Attitudes towards recovery were assessed using the Japanese version of the seven-item Recovery Attitudes Questionnaire (RAQ), originally developed by Borkin et al. (24) and translated and validated by Chiba et al. (25). The RAQ assesses beliefs about the possibility and nature of recovery from mental illness and includes two subscales: “Recovery is possible and needs faith” (Belief) and “Recovery is difficult and differs among people” (Difficult). Items were rated on a five-point Likert scale ranging from “strongly disagree” to “strongly agree.” In this study, the Cronbach’s α coefficients were 0.830 for the total score, 0.714 for the belief subscale, and 0.765 for the difficulty subscale.

#### 2.3.7 Job stressor

Job stressor was measured using the PNJSS, developed by Yada et al. (26). The Psychiatric Nurse Job Stressor Scale (PNJSS) includes 22 items across four domains: Psychiatric Nursing Ability, Attitude of Patients, Attitude Toward Nursing, and Communication. Items were rated using a 0–100-point visual analogue scale, with higher scores indicating greater perceived stressor. The Cronbach’s α coefficients in this study were 0.823 for the total score, 0.882 for Psychiatric Nursing Ability, 0.808 for Attitude of Patients, 0.630 for Attitude Toward Nursing, and 0.758 for Communication.

#### 2.3.8 Moral distress

Moral distress was measured using the MDS-P, developed by Ohnishi et al. (14). This scale was adapted from the Moral Distress Scale for critical care nurses by Corley et al. (27). It comprises 15 items divided into three subscales: Unethical conduct by caregivers (Unethical behavior), Low staffing (Low staffing), and Acquiescence to patients’ rights violations (Tolerance violation). Items were rated on a seven-point Likert scale ranging from 0 (“none”) to 6 (“a great extent”). In the present study, the Cronbach’s α coefficients were 0.861 for the total score, 0.786 for unethical conduct, 0.731 for low staffing, and 0.689 for rights violations.

### 2.4 Statistical analysis

All analyses were conducted using R software (version 4.3.0; R Development Core Team, Vienna, Austria). First, descriptive statistics were calculated to summarize the prevalence of each of the 32 abusive behaviors. For this purpose, responses were dichotomized based on frequency: “No Engagement” (Never [1]) and “Any Engagement” (Once [2] to Weekly or more [6]). Participants were classified into the Any Engagement group if they reported having engaged in at least one of the 32 behaviors. However, during preparation for logistic regression analysis, we found that some categories of explanatory variables included no participants in one of the outcome groups (i.e., n = 0). To address this issue, we applied an alternative classification based on frequency. In this revised scheme, responses were dichotomized into Low-Frequency Engagement (Never [1] or Once [2]) and High-Frequency Engagement (Rarely [3] to Weekly or more [6]). Participants were classified into the High-Frequency group if they endorsed any of the 32 behaviors at that frequency level. Pearson correlation coefficients were then calculated to examine relationships among the total scores for moral sensitivity (J-MSQ2018), recovery attitudes (RAQ), ethical climate (J-HECS), and job stressor (PNJSS).

To further explore factors associated with abusive behaviors, we conducted a logistic regression analysis using the High-Frequency Engagement classification as the dependent variable. Independent variables included gender, nursing qualification, years of clinical experience, ward type, experience of workplace violence, and total scores on the J-MSQ2018, RAQ, J-HECS, and PNJSS. The significance level was set at p < 0.05. There was no missing data, as the online questionnaire required complete responses.

## 3 Results

### 3.1 Prevalence of abusive behaviors

Among the 203 psychiatric nursing staff who completed the survey, 87.1% (n = 177) reported having engaged in at least one abusive behavior in the past year (classified as the “Any Engagement group”). Of all participants, 77.8% (n = 158) reported engaging in at least one abusive behavior at a frequency of “Rarely” or higher (classified as the “High-Frequency group”). The percentages of reported abuse under the two classification categories are presented in **Table 1**. The most commonly reported abusive behaviors, based on High-Frequency Engagement, were “ignoring or rejecting the patient” (53.2%), “covert medication administration” (37.4%), and “forcing patients to urinate or defecate in diapers” (33.0%). Other frequently cited behaviors included “words or actions that undermine dignity” (24.6%) and “discussing private matters in front of others” (24.1%). Although infrequent, “physical violence against the patient” was reported by 3.9% of participants, and sexual misconduct by 1.5% (**Table 1**).

**Table 1:** Prevalence of self-reported abusive behaviors by occurrence type (N = 203).

Abuse item	Any engagement (%)	High-frequency group (%)
Ignoring or Rejecting the Patient	125 (61.6)	108 (53.2)
Covert Medication Administration	88 (43.3)	76 (37.4)
Forcing Patients to Urinate or Defecate in Diapers	87 (42.9)	67 (33.0)
Words or Actions that Undermine Dignity	71 (35.0)	50 (24.6)
Providing Toileting or Dressing Assistance in Public View	59 (29.1)	50 (24.6)
Discussing Private Matters in Front of Others	63 (31.0)	49 (24.1)
Using Insulting or Derogatory Nicknames	58 (28.6)	47 (23.2)
Threatening or Intimidating Behavior	60 (29.6)	44 (21.7)
Withholding Environmental Maintenance Without Medical Reason	51 (25.1)	44 (21.7)
Inadequate Informed Consent for Treatment	45 (22.2)	34 (16.7)
Intimidating or Punitive Response to Bathing Refusal	49 (24.1)	30 (14.8)
Punitive Use of Restraint	32 (15.8)	26 (12.8)
Withholding Personal Hygiene Care Without Medical Reason	31 (15.3)	26 (12.8)
Excessive Force in Handling Patients	35 (17.2)	25 (12.3)
Neglecting Patient-to-Patient Violence or Threats	34 (16.7)	25 (12.3)
Neglecting Necessary Care During Restraint	32 (15.8)	25 (12.3)
Ignoring Financial Conflicts Among Patients	33 (16.3)	24 (11.8)
Intimidating or Punitive Response to Food Refusal	35 (17.2)	22 (10.8)
Initiating or Prolonging Restraint for Staff Convenience	26 (12.8)	18 (8.9)
Administering Medication as Chemical Restraint for Staff Convenience	25 (12.3)	18 (8.9)
Punitive Medication Administration	24 (11.8)	17 (8.4)
Use of excessive physical force during restraint	29 (14.3)	15 (7.4)
Withholding Toileting Assistance Without Medical Reason	20 (9.9)	14 (6.9)
Withholding Eating Assistance Without Medical Reason	16 (7.9)	14 (6.9)
Punitive Restrictions on Patient Property	15 (7.4)	12 (5.9)
Punitive Response to Incontinence	19 (9.4)	11 (5.4)
Improper Use of Restraint	16 (7.9)	9 (4.4)
Ignoring Noticeable Patient Abnormalities	16 (7.9)	9 (4.4)
Physical Violence Against the Patient	12 (5.9)	8 (3.9)
Unjustified Management of Patient Property	9 (4.4)	6 (3.0)
Unauthorized Use of Patient Property	6 (3.0)	4 (2.0)
Inappropriate Sexual Contact or Remarks	6 (3.0)	3 (1.5)

“Any Engagement” refers to participants who reported having engaged in the behavior at least once. “High-Frequency group” refers to participants who reported having engaged in the behavior at a frequency of “Rarely” or more.

### 3.2 Participant characteristics and psychological measures

As shown in **Table 2**, several statistically significant differences were observed between participants in the High-Frequency group (n = 158) and those in the Low-Frequency group (n = 45). In particular, the distribution of nursing qualifications differed significantly between the groups (p = 0.044). Those in the High-Frequency group also had fewer years of clinical experience (mean = 14.1 vs. 19.0 years, p = 0.010). A significant difference was also found in ward type (p = 0.039), with a higher proportion of High-Frequency participants working in locked wards. Additionally, workplace violence was significantly more common among participants in the High-Frequency group (79.7% vs. 48.9%, p < 0.001). No other significant group differences were identified.

**Table 2:** Comparison of participant characteristics by frequency of engagement in abusive behaviors (N = 203).

Variable	Total	Low-Frequency Group (n= 45)	High-Frequency Group (n = 158)	p-value
	Mean/n (SD/%)	Mean/n (SD/%)	Mean/n (SD/%)	
Age (y)	42.0 (12.1)	44.9 (12.7)	41.2 (11.79)	0.071
Sex				
Female	131 (64.5)	31 (68.9)	100 (63.3)	0.606
Male	72 (35.5)	14 (31.1)	58 (36.7)	
Qualification				
Registered Nurse	141 (69.5)	27 (60.0)	114 (72.2)	0.044
Enrolled Nurse	35 (17.2)	7 (15.6)	28 (17.7)	
Nurse Aid	27 (13.3)	11 (24.4)	16 (10.1)	
Employment				
Full Time	196 (96.6)	41 (91.1)	155 (98.1)	0.071
Part Time	7 (3.4)	4 (8.9)	3 (1.9)	
Clinical Experience (y)	15.1 (10.8)	19.0 (11.2)	14.1 (10.5)	0.010
Psychiatric Experience (y)	10.9 (8.3)	12.2 (8.7)	10.5 (8.2)	0.226
Ward function				
Acute Ward	62 (30.5)	13 (28.9)	49 (31.0)	0.929
Long-stay Ward	141 (69.5)	32 (71.1)	109 (69.0)	
Ward type				
Locked	176 (86.7)	34 (75.6)	142 (89.9)	0.039
Open	21 (10.3)	9 (20.0)	12 (7.6)	
Mixed	6 (3.0)	2 (4.4)	4 (2.5)	
Workplace Violence	148 (72.9)	22 (48.9)	126 (79.7)	<0.001
Moral sensitivity (J-MSQ 2018)				
Total Score	36.91 (7.85)	37.69 (9.95)	36.68 (7.17)	0.450
Moral Strength Score	10.33 (2.87)	10.53 (3.45)	10.28 (2.69)	0.600
Moral Awareness Score	14.92 (3.46)	15.40 (4.20)	14.78 (3.22)	0.289
Moral Responsibility Score	11.66 (3.25)	11.76 (3.61)	11.63 (3.15)	0.815
Ethical climate (J-HECS)				
Total Score	59.9 (12.9)	63.0 (16.3)	59.0 (11.7)	0.065
Colleague Score	14.7 (3.2)	15.4 (4.1)	14.5 (2.9)	0.115
Supervisor Score	11.3 (3.1)	11.1 (3.6)	11.4 (3.0)	0.642
Hospital Score	12.3 (3.6)	13.1 (4.2)	12.1 (3.4)	0.097
Patient Score	9.8 (2.1)	10.4 (2.7)	9.7 (1.9)	0.052
Doctor Score	11.7 (3.7)	13.0 (3.8)	11.3 (3.5)	0.005
Job stressor (PNJSS)				
Psychiatric Nursing Ability Score	42.2 (15.4)	39.8 (15.0)	42.9 (15.5)	0.229
Attitude of Patients Score	38.3 (18.4)	34.6 (19.7)	39.4 (18.0)	0.126
Attitude Toward Nursing Score	57.2 (15.8)	52.2 (17.4)	58.6 (15.1)	0.016
Communication Score	49.7 (23.0)	49.5 (26.4)	49.7 (22.1)	0.964
Recovery attitude (RAQ)				
Total Score	26.1 (4.8)	25.2 (6.3)	26.4 (4.3)	0.160
Belief Score	14.2 (2.9)	14.0 (3.5)	14.2 (2.7)	0.743
Difficulty Score	12.0 (2.4)	11.2 (3.1)	12.2 (2.1)	0.014
Moral Distress (MDS-P)				
Total Score	44.2 (19.9)	37.7 (26.1)	46.1 (17.4)	0.012
Unethical Behavior Score	15.4 (10.0)	13.3 (13.0)	16.1 (8.9)	0.097
Low Staffing Score	19.0 (7.6)	16.3 (9.4)	19.7 (6.9)	0.007
Tolerance Violation Score	9.8 (6.3)	8.1 (7.5)	10.3 (5.9)	0.042

The High-Frequency group was defined as participants who endorsed at least one of the 32 abusive behavior items at a frequency of “Rarely” (i.e., once every 6 months) or more.

The Low-Frequency group included those who responded “Never” or “Once.”

p-values were calculated using independent samples t-tests or chi-square tests, as appropriate.

SD, standard deviation; J-MSQ 2018, Japanese version of the Moral Sensitivity Questionnaire 2018; J-HECS, Japanese version of the Hospital Ethical Climate Survey; RAQ, Japanese version of the Recovery Attitudes Questionnaire; PNJSS, Psychiatric Nurse Job Stressor Scale; MDS-P, Moral Distress Scale for Psychiatric Nurses.

Regarding psychological measures, no significant group differences were observed in the total scores for the J-MSQ2018, J-HECS, or RAQ. However, the High-Frequency group scored significantly higher on the RAQ “difficulty” subscale (p = 0.014) and the PNJSS Attitude Toward Nursing subscale (p = 0.016). In terms of moral distress, the High-Frequency group reported significantly higher total scores on the MDS-P (mean = 46.1 vs. 37.7, p = 0.012), particularly for the “Low staffing” (p = 0.007) and Tolerance violation (p = 0.042) subscales (**Table 2**).

Pearson correlation analysis revealed that the J-MSQ2018 score was positively correlated with the RAQ (r = 0.60, p < 0.01) and J-HECS (r = 0.28, p < 0.01) scores. The RAQ and J-HECS scores were also positively correlated (r = 0.32, p < 0.01). In contrast, the PNJSS score was negatively correlated with the J-MSQ2018 (r = –0.29, p < 0.01) and J-HECS (r = –0.35, p < 0.01) scores. No significant correlation was observed between the PNJSS and RAQ scores (r = –0.10) (**Table 3**).

**Table 3:** Correlation matrix of moral sensitivity, recovery attitudes, ethical climate, and job stressors scores.

	J-MSQ2018	J-HECS	RAQ	PNJSS
J-MSQ2018	–	0.28*	0.60*	-0.29*
J-HECS	0.28*	–	0.32*	-0.35*
RAQ	0.60*	0.32*	–	-0.10
PNJSS	-0.29*	-0.35*	-0.10	–

Data are instrument total scores.

*p < 0.01.

J-MSQ 2018, Japanese version of the Moral Sensitivity Questionnaire 2018; J-HECS, Japanese version of the Hospital Ethical Climate Survey; RAQ, Japanese version of the Recovery Attitudes Questionnaire; PNJSS, Psychiatric Nurse Job Stressor Scale.

### 3.3 Factors associated with high-frequency engagement in abusive behaviors

Logistic regression analysis identified several factors associated with self-reported frequency of abusive behaviors (**Table 4**). Experience of workplace violence and RAQ scores were significantly associated with higher risk of high-frequency abuse (adjusted odds ratio [AOR] = 3.37, 95% confidence interval [CI]: 1.49–7.72; AOR = 1.17, 95% CI: 1.05–1.32). In contrast, higher moral sensitivity scores (J-MSQ2018) and longer clinical experience were associated with lower frequency of abuse (AOR = 0.92, 95% CI: 0.86–0.99; AOR = 0.95, 95% CI: 0.92–0.99). No significant associations were found for sex, nursing qualification, ward type, PNJSS scores, or J-HECS scores.

**Table 4:** Logistic regression analysis of factors associated with high-frequency engagement in abusive behaviors.

Variable	Adjusted OR	95% CI
Sex (Ref: Female)	1.27	0.54 – 3.13
Nursing Qualification (Ref: No Qualification)	2.39	0.81 – 6.94
Clinical Experience	**0.95**	**0.92 – 0.99**
Ward Type (Ref: Open)	2.82	0.98 – 8.03
Workplace Violence	**3.37**	**1.49 – 7.72**
Moral Sensitivity (J-MSQ 2018)	**0.92**	**0.86 – 0.99**
Ethical Climate (J-HECS)	**0.97**	**0.93 – 1.00**
Recovery Attitudes (RAQ)	**1.17**	**1.05 – 1.32**
Job Stressor (PNJSS)	1.01	0.97 – 1.06

High-Frequency Engagement was defined as endorsing at least one of the 32 abusive behavior items at a frequency of “Rarely” (i.e., once every 6 months) or more.

Adjusted odds ratios (ORs) were obtained from a multivariable logistic regression model.

Statistical significance was determined on the basis of 95% confidence intervals (CI).

J-MSQ 2018, Japanese version of the Moral Sensitivity Questionnaire 2018; J-HECS, Japanese version of the Hospital Ethical Climate Survey; RAQ, Japanese version of the Recovery Attitudes Questionnaire; PNJSS, Psychiatric Nurse Job Stressor Scale.

Bold values indicate statistical significance at p < 0.05.

## 4 Discussion

This study investigated the prevalence and determinants of abusive behaviors by nursing staff in psychiatric hospitals in Japan. A high proportion of participants reported engaging in at least one abusive behavior in the past year (88.2%). Furthermore, 77.8% reported engaging in at least one behavior at a frequency of “Rarely” or more, indicating repeated or habitual engagement rather than isolated incidents. Psychological abuse and human rights violation were the most frequently reported. Logistic regression analysis revealed that experience of workplace violence and stronger recovery-oriented attitudes were associated with increased odds of abusive behaviors, whereas greater moral sensitivity and longer clinical experience were associated with decreased odds. These findings highlight the complex interplay of individual, professional, and environmental factors in the occurrence of abuse, and suggest the need for multifaceted preventive strategies that address not only workplace conditions and ethical competencies, but also professional attitudes and beliefs about recovery. These associations should be interpreted with caution, as it is also possible that exposure to abusive environments may, in turn, affect nurses’ moral sensitivity, recovery orientation, or professional attitudes toward recovery.

Patient surveys have reported that between 10% and nearly 50% of individuals with psychiatric hospitalization histories have experienced abuse, although the rates vary depending on the type of abuse assessed (28–30). In contrast, the remarkably high prevalence observed in the present study—87.1% of participants reporting any abusive behaviors, and 77.8% classified as High-Frequency Engagement—calls for careful interpretation. Several factors may contribute to these findings. First, the broad conceptualization of abuse adopted in this study, encompassing not only overt physical or sexual violence but also psychological neglect, coercive practices, and inappropriate behaviors, likely increased the reported rates. Second, the anonymous, self-administered online survey format may have encouraged more honest responses, especially regarding ethically sensitive topics. The rates of the most frequently reported behaviors, such as “ignoring or rejecting the patient,” “covert medication administration,” and “forcing patients to use diapers,” suggest that many abusive actions are not overtly violent but embedded in routine practices and institutional norms. These findings highlight the thin line between standard care procedures and potentially abusive conduct in psychiatric settings. Indeed, a study of individuals who had been hospitalized in psychiatric facilities in Japan found that nearly 90% reported not receiving adequate explanations regarding their admission or treatment (31). In contrast, physical and sexual abuse were reported at much lower rates, which may reflect their social and legal unacceptability or hesitancy to report such behaviors, even anonymously. The gap between the most prevalent behaviors and those identified by staff as the most problematic suggests potential normalization of certain coercive practices (8), underscoring the need for critical reflection on what constitutes ethical care in psychiatric wards.

The present study identified a significant association between experiences of workplace violence and engagement in abusive behaviors among psychiatric nursing staff. This finding aligns with prior research indicating that nurses exposed to violence are more likely to experience emotional exhaustion and compassion fatigue, and to subsequently display aggression towards patients (32). However, this relationship should not be interpreted in terms of a simple causal pathway. Psychiatric care environments inherently involve coercive interventions such as seclusion, physical restraint, and involuntary treatment—practices often perceived by patients as abusive (18). Our own findings also indicated that abuse frequently occurs in situations involving coercion, including forced care procedures. Such inappropriate coercive practices can be experienced by patients as traumatic events and may hinder therapeutic engagement and recovery (33, 34). These coercive measures or abusive behaviors may provoke aggression from patients, leading to a cyclical dynamic in which staff use further coercive or abusive strategies in response (8, 35). Thus, the relationship between patient violence and staff-perpetrated abuse may be bidirectional and embedded within a broader system of institutionalized coercion. Therefore, addressing abuse in psychiatric settings requires not only individual-level interventions such as training and ethical development but also systemic changes that mitigate structural violence and reduce reliance on coercive practices.

Another notable finding was the paradoxical association between stronger recovery-oriented attitudes and a higher likelihood of reporting abusive behaviors. While recovery-oriented care is typically associated with patient-centered and respectful approaches (36, 37), qualitative research has indicated that nurses who deeply commit to recovery principles may experience intensified frustration when systemic or clinical barriers obstruct their efforts to realize those ideals (38, 39). For example, limited institutional resources, rigid protocols, or persistent treatment-resistant symptoms may hinder efforts to promote autonomy and recovery, thereby leading to emotional strain and potentially maladaptive responses. Another possible interpretation involves the role of self-report bias. Person-centered care—a concept closely related to recovery-oriented practice—has been associated with heightened moral sensitivity (40), which, in turn, has been linked to increased recognition of ethical issues in clinical settings (41). Consequently, nurses who value recovery principles may also demonstrate heightened ethical awareness and self-reflective capacity, making them more attuned to ethically troubling behaviors and more inclined to acknowledge them. This interpretation is further supported by the observed positive association between recovery-oriented attitudes and moral sensitivity in the present study. In contrast, nurses with weaker recovery orientations might lack this moral sensitivity, potentially leading to underreporting of abusive behavior. These possibilities underscore the interpretive complexity of self-reported abuse and highlight the importance of complementing quantitative data with qualitative or observational methodologies in future research.

Furthermore, although the ethical climate did not show a significant association with abusive behaviors, participants in the high-frequency group reported significantly lower scores on the subscale related to relationships with psychiatrists. Given that abusive behaviors were commonly observed in coercive care situations, it is possible that difficulties in voicing nursing perspectives to psychiatrists may contribute to moral burden and, indirectly, to ethically inappropriate practices (42). Future studies should therefore examine not only peer relationships among nurses but also interprofessional dynamics and the broader ethical climate within psychiatric organizations.

The findings of this study have broader implications for psychiatric nursing practice, organizational policy, and ethical education. The high prevalence of reported abuse, including both psychological and institutional forms, underscores the need to re-examine routine care practices through a human rights and recovery-oriented lens (43). Prior research has documented widespread incidents and complaints related to coercive practices, highlighting the systemic nature of such issues within psychiatric settings (44). Interventions aimed at reducing abuse must therefore extend beyond individual-level strategies to address systemic and environmental contributors, such as chronic understaffing, inadequate ethical training, and insufficient institutional support. From a theoretical perspective, the results support a systems-based understanding of moral distress and abuse, wherein ethical lapses are not merely personal failings but emerge from structural and contextual constraints. Recent theoretical work has emphasized how structural, cultural, and organizational forces intersect to create conditions in which abuse becomes possible, calling for a transformation of nursing practice through empowerment and systemic change (45). Clinically, these findings advocate for integrated staff support systems, including regular ethical debriefings, interdisciplinary ethics rounds, and mechanisms for reporting and addressing moral concerns (46, 47). At the policy level, the implementation of institutional accountability frameworks and routine ethical audits may serve as preventive mechanisms (2). These multifaceted approaches are essential to fostering a safe and ethically sound care environment in psychiatric settings.

### 4.1 Strengths and limitations

Several limitations of the present study should be acknowledged when interpreting the findings. First, the use of a cross-sectional, self-reported survey design limits causal inference and introduces the possibility of recall and social desirability biases. Because the relationships between variables were examined at a single point in time, the directionality of associations cannot be determined, and reverse causality cannot be ruled out. Although the anonymity of the online format may have encouraged honest reporting, the sensitive nature of the topic may still have led to underreporting or selective disclosure. Although questionnaires were sent according to the requests of nursing managers at each facility, the exact number actually distributed is unknown, which may have introduced response bias. Nevertheless, even if the prevalence was underestimated, the results clearly reveal the existence of abusive practices and strongly indicate the need for preventive measures. In addition, because this study adopted a broad definition of abuse, the overall prevalence was high. As a result, we reclassified the frequency of abusive behaviors and examined the associated factors. In future research, it will be important to investigate these factors in greater detail for each specific type of abuse. Second, the participating hospitals were all engaged in abuse prevention initiatives, which may limit generalizability to institutions with different organizational cultures or priorities. Depending on how these initiatives influenced staff awareness and reporting behaviors, the prevalence of abuse could have been either underestimated (if staff became more cautious in their self-reports) or overestimated (if awareness increased their sensitivity to inappropriate behaviors). Third, although the original abuse questionnaire was developed on the basis of prior literature and preliminary qualitative interviews, it remains a newly constructed instrument. As such, it may not fully capture the breadth or nuances of abusive behaviors occurring in real-world clinical settings. In the present study, the internal consistency of the 32-item scale was good (Cronbach’s α = 0.90); however, formal validation procedures such as factor analysis and construct validity testing were not conducted. Future studies should incorporate longitudinal and mixed-methods designs to examine the dynamics of abuse over time and explore contextual factors in greater depth. Triangulating self-report data with observational or patient-reported measures may also improve the robustness of the findings. Fourth, although this study examined individual and attitudinal factors such as moral sensitivity, recovery orientation, and occupational stress, organizational and contextual factors (e.g., staffing levels, nurse-to-patient ratios, and ward climate) were not assessed. However, a qualitative study of psychiatric nursing managers’ perspectives (42) suggested that organizational and cultural factors may contribute to abusive behaviors. Future research should integrate both individual- and organizational-level data to gain a deeper understanding of how institutional environments influence the occurrence and prevention of abuse. Despite these limitations, the present study contributes important empirical evidence on the prevalence and correlates of staff-perpetrated abuse in psychiatric settings and underscores the need for ethically grounded, system-level interventions to foster safer and more respectful care environments.

## 5 Clinical implications and conclusion

This study investigated the prevalence of and factors associated with abusive behaviors by nursing staff in psychiatric hospitals in Japan. A substantial proportion of nurses reported engaging in various forms of abuse such as neglect, covert medication, and coercive care practices. Importantly, abusive behavior was associated with individual, professional, and contextual factors, including workplace violence, moral sensitivity, recovery-oriented attitudes, and clinical experience. These results underscore the complexity of abuse in psychiatric settings and highlight the need for multifaceted preventive strategies. Addressing abuse may require not only improving working conditions and reducing staff exposure to violence, but also enhancing ethical awareness and fostering organizational environments that support humane and recovery-oriented care. Future research should further investigate the mechanisms underlying staff-perpetrated abuse and develop evidence-based interventions that can be integrated into psychiatric care systems. Ultimately, promoting ethical practice and protecting patient dignity must remain central goals in psychiatric nursing.

The findings of this study highlight the urgent need for psychiatric healthcare systems to recognize and address abusive behaviors by nursing staff. Many of the reported behaviors—such as neglect, verbal mistreatment, and covert coercion—are deeply embedded in daily routines and may go unrecognized without critical ethical reflection. Clinical practice must therefore integrate ongoing education on ethical care, promote moral sensitivity, and establish organizational cultures that prioritize patient dignity and recovery-oriented approaches. Moreover, addressing staff exposure to workplace violence and ensuring adequate staffing are essential steps in mitigating conditions that may be linked to abusive behaviors. Routine ethical audits, structured debriefings, and open communication channels may further support staff in delivering safe, respectful, and ethically sound care.

## Data Availability

The raw data supporting the conclusions of this article will be made available by the authors, without undue reservation.

## Supplementary material

Supplementary File 1 Interview guide used for the semi-structured interviews with nursing managers. The guide outlines the key question areas related to the understanding of factors contributing to abuse in psychiatric hospital settings.
