# Supplementary material for: Nurse-perpetrated abuse in Japanese psychiatric hospitals: a cross-sectional study of prevalence and correlates
Source: Front Psychiatry. 2025 Nov 24;16:1624859. doi: 10.3389/fpsyt.2025.1624859 (PMC12682874; doi:10.3389/fpsyt.2025.1624859)
Supplement: Supplementary File 1 — Interview guide used for the semi-structured interviews with nursing managers. The guide outlines the key question areas related to the understanding of factors contributing to abuse in psychiatric hospital settings. [file DataSheet1.pdf]

Supplementary Table S1. Original 32-item Abuse Questionnaire

Overview (English):

This 32-item questionnaire was developed by the research team to assess self-reported abusive or inappropriate behaviors by nursing staff toward patients in psychiatric hospitals.

Each item represents a potentially inappropriate or abusive behavior observed in clinical practice, encompassing six domains:

(1) *Psychological abuse* (e.g., verbal threats, neglect, humiliation)

(2) *Physical abuse* (e.g., excessive restraint, unnecessary physical force)

(3) *Sexual misconduct* (e.g., unwanted contact or remarks)

(4) *Economic abuse* (e.g., misuse or restriction of patients' belongings)

(5) *Neglect of care* (e.g., failure to assist with meals, hygiene, or elimination)

(6) *Human rights violations* (e.g., violation of privacy, coercive or punitive care)

Respondents rated how frequently they had personally engaged in each behavior during the past 12 months using a 6-point Likert scale:

1 = Never, 2 = Once, 3 = Rarely (once every 6 months), 4 = Occasionally (every 2–3 months), 5 = Monthly, 6 = Weekly or more.

Original Japanese version:

虐待（不適切行為）について

以下に示すものは、精神科病棟で報告されている不適切行為の例です。以下の項目について、過去12か月の間で、あなた自身が行った行為について、最も近い数字を選択してください。

※他の職員の行為を目撃した経験は含みません。

		1：全くなかった	2：1回あった	3：時々あった（6ヶ月に1回以下）	4：2・3ヶ月に1回程度あった	5：月に1回程度あった	6：週に1回以上あった
1	患者を無視、または拒絶した（ナースコールを含む）	1	2	3	4	5	6
2	患者を脅迫、または威圧するような関わりをした	1	2	3	4	5	6

3	患者を呼び捨てにしたり、あだ名で呼んだり、侮辱的な呼びかけを行った（例：’おい’、’お前’など）	1	2	3	4	5	6
4	患者の尊厳を傷つける言葉や態度で関わった（暴言、怒鳴る・叱る、命令など）	1	2	3	4	5	6
5	懲罰的に薬を与薬した、あるいは与薬を脅しに使用した	1	2	3	4	5	6
6	患者に分からない方法で薬を与薬した（食事に混ぜるなど）	1	2	3	4	5	6
7	懲罰的に行動制限を行った、あるいは行動制限を脅しに使用した	1	2	3	4	5	6
8	適切な手順や方法を用いずに行動制限を行った	1	2	3	4	5	6
9	行動制限中に適切な関わりを行わなかった（巡視を行わなかったなど）	1	2	3	4	5	6
10	患者を抑止するために必要以上の力で抑制した	1	2	3	4	5	6
11	患者を必要以上の力で引きずったり、介助した	1	2	3	4	5	6
12	患者に対して身体的暴力をふるった	1	2	3	4	5	6
13	患者に対して不必要な性的接触、言動を行った	1	2	3	4	5	6
14	患者間の金銭トラブルを黙認した	1	2	3	4	5	6
15	患者の財産・私物（間食を含む）を患者の同意なく使用した	1	2	3	4	5	6
16	患者の財産・私物（間食を含む）を相当の理由もなく管理した	1	2	3	4	5	6
17	患者の財産・私物（間食を含む）を懲罰的理由で制限した	1	2	3	4	5	6
18	排泄の失敗に対して懲罰的な態度をとった	1	2	3	4	5	6
19	トイレへ誘導せずオムツ等の中に排泄するよう言った	1	2	3	4	5	6
20	患者を他者の目の届く場所、状況で排泄・更衣の援助を行った	1	2	3	4	5	6
21	治療上の理由なく排泄の援助を行わなかった	1	2	3	4	5	6

22	個室等で話すようなプライバシーにかかわる話を他者の前でした（他患者のケア中も含む）	1	2	3	4	5	6
23	患者に対して、病状や治療についての十分なインフォームド・コンセントを行わなかった、あるいは行われていない状況を放置した	1	2	3	4	5	6
24	食事を拒否する患者に対して威圧的、懲罰的に関わった	1	2	3	4	5	6
25	治療上の理由なく食事の援助を<u>行わなかった</u>（患者が希望する飲水の提供を行わないことも含む）	1	2	3	4	5	6
26	入浴を拒否する患者に対して、威圧的、懲罰的に関わった	1	2	3	4	5	6
27	治療上の理由なく清潔の援助を<u>行わなかった</u>	1	2	3	4	5	6
28	治療上の理由なく環境整備を<u>行わなかった</u>	1	2	3	4	5	6
29	患者間で身体的暴力、脅迫、言葉による侮辱や暴言が行われているところを放置した	1	2	3	4	5	6
30	患者の異常を知りながら放置した	1	2	3	4	5	6
31	医療者側の都合で薬を与薬し動きを抑制した	1	2	3	4	5	6
32	医療者側の都合で隔離や身体拘束を開始、継続した	1	2	3	4	5	6
